# The Saturation Effect of Body Mass Index on Bone Mineral Density for People Over 50 Years Old: A Cross-Sectional Study of the US Population

**DOI:** 10.3389/fnut.2021.763677

**Published:** 2021-10-15

**Authors:** Ming Ma, Zhiwei Feng, Xiaolong Liu, Gengxin Jia, Bin Geng, Yayi Xia

**Affiliations:** ^1^The Second School of Clinical Medical, Lanzhou University, Lanzhou, China; ^2^Orthopaedics Key Laboratory of Gansu Province, Lanzhou, China; ^3^Department of Orthopaedics, Lanzhou University Second Hospital, Lanzhou, China

**Keywords:** Body Mass Index, Bone Mineral Density, femur, lumbar spine, National Health and Nutrition Examination Survey (NHANES)

## Abstract

**Background:** Previous studies had revealed that Body Mass Index (BMI) positively affected Bone Mineral Density (BMD). However, an excessively high BMI was detrimental to health, especially for the elderly. Moreover, it was elusive how much BMI was most beneficial for BMD in older adults to maintain.

**Objective:** To investigate whether there was a BMI saturation effect value that existed to maintain optimal BMD.

**Methods:** A cross-sectional study was conducted using the datasets of the National Health and Nutrition Examination Survey (NHANES) 2005–2006, 2007–2008, 2009–2010, 2013–2014, and 2017–2018. After adjusting for covariates, an analysis of the association between BMI and BMD in different femoral regions (Total femur, Femoral neck, Trochanter, Intertrochanter, and Ward's triangle) and lumbar spine regions (Total spine, L1, L2, L3, and L4) in the whole population was performed using the multivariate linear regression models, smoothing curve fitting, and saturation effects analysis models. Then, subgroup analyses were performed according to gender, age, and race.

**Results:** A total of 10,910 participants (5,654 males and 5,256 females) over 50 years were enrolled in this population-based study. Multivariate linear regression analyses in the population older than 50 years showed that BMI was positively associated with femoral BMD and lumbar spine BMD (*P* < 0.001, respectively). Smoothing curve fitting showed that the relationship between BMI and BMD was not simply linear and that a saturation value existed. The saturation effect analysis showed that the BMI saturation value was 26.13 (kg/m^2^) in the total femur, 26.82 (kg/m^2^) in the total spine, and showed site-specificity in L1 (31.90 kg/m^2^) and L2 (30.89 kg/m^2^). The saturation values were consistent with the whole participants in males, while there was high variability in the females. BMI saturation values remained present in subgroup analyses by age and race, showing specificity in some age (60–70 years old) groups and in some races.

**Conclusions:** Our study showed a saturation value association between BMI and BMD for people over 50 years old. Keeping the BMI in the slightly overweight value (around 26 kg/m^2^) might reduce other adverse effects while obtaining optimal BMD.

## Introduction

Osteoporosis was a common disease of the skeletal system that imposed a substantial economic burden on society (1, 2) and posed a risk of fracture in the elderly (3, 4). As the most common clinical examination index, BMD was the gold standard for assessing osteoporosis (5). Obesity was one of the major public health issues to face in today's society, especially in the United States, with the highest adult obesity prevalence globally (6). Similarly, BMI was used in clinical situations to assess overweight and obesity (7).

Most of the available studies showed a positive association between BMI and BMD. A study in a 70-year-old population showed that patients with high BMI had a higher BMD and a lower risk of osteoporosis than those with a normal BMI (8). In older men and postmenopausal women, an increase in BMI was accompanied by BMD (9–11). However, an excessive BMI caused various other systemic diseases and complications such as hypertension (12), coronary heart disease (13), and type 2 diabetes (14). Possible mechanisms involved in the above diseases were the activation of the sympathetic nervous system (15), damage to the vascular endothelium (16), or insulin resistance (17).

Therefore, it was essential to strike a balance between BMI and BMD. However, existing researches were unclear exactly how much BMI was most beneficial for BMD while reducing the occurrence of other obesity-related complications. Therefore, we investigated the association between BMI and BMD in this work using a cross-sectional population survey sample from the NHANES (18) database for participants aged above 50 years, representing all regions and major ethnicities in the United States. We hypothesize that there was a saturation value of BMI and that keeping BMI at this value would result in an optimal balance between BMI and BMD.

## Materials and Methods

### Data Sources

The present study used the data sets from the NHANES database to carry out a cross-sectional study. NHANES is a national health information source to collect the health and nutrition of adults and children in the USA. The NHANES project had approved by the National Center of Health Statistics (NCHS) Research Ethics Review Board (ERB), and every willing participant signed a consent document before starting. (NCHS IRB/ERB Protocol Number: Protocol #2005-06, Continuation of Protocol #2005-06, Continuation of Protocol #2011-17, Protocol #2018-01, https://www.cdc.gov/nchs/nhanes/irba98.htm).

### Participants Selected

**Figure 1:**
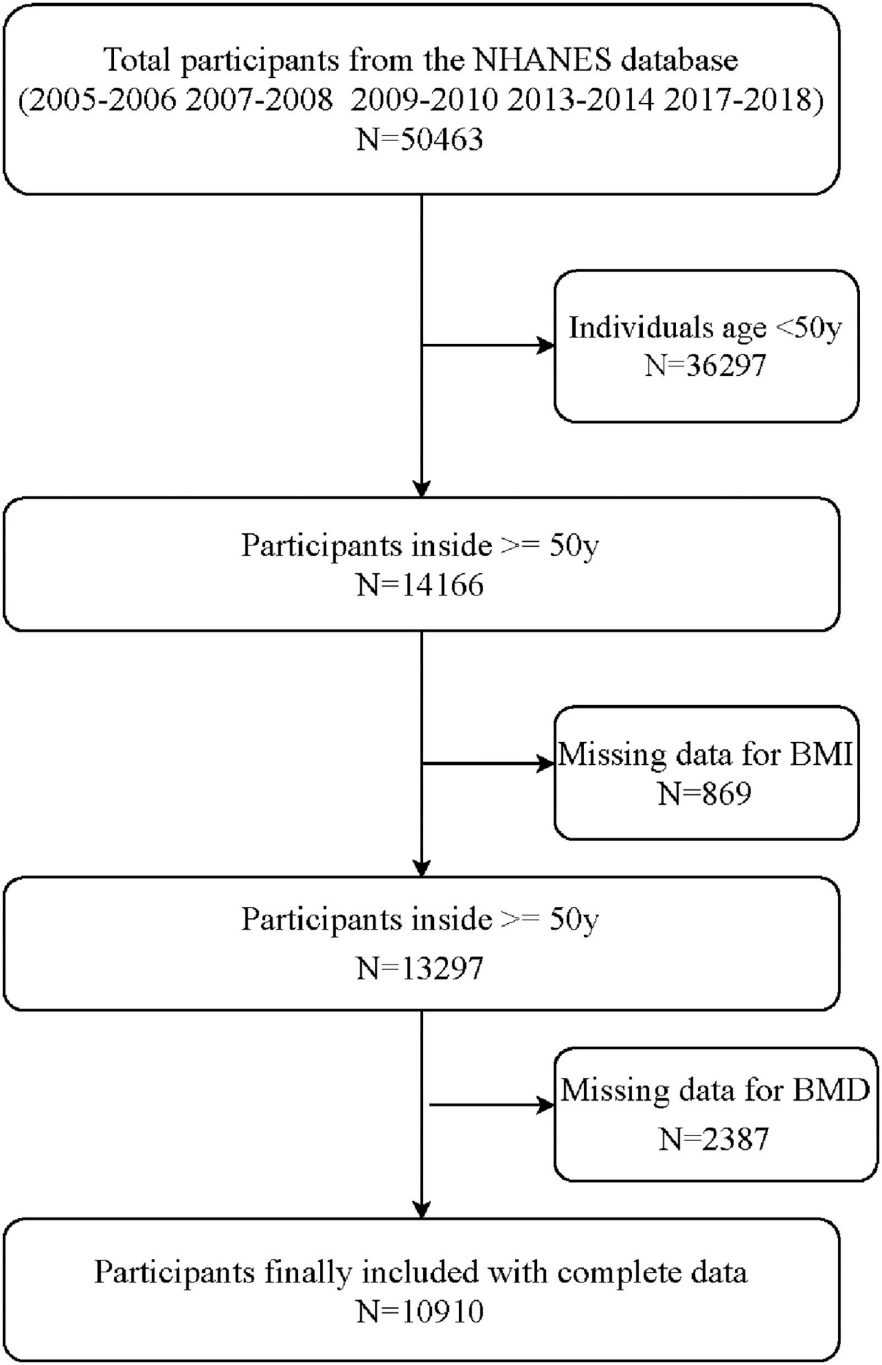
Flow chart of participants selected from the NHANES database.

Data sets were used from NHANES 2005–2006, NHANES 2007–2008, NHANES 2009–2010, NHANES 2013–2014, and NHANES 2017–2018 because femur BMD data were only collected the above period. Before the study began, the following people were excluded from BMD testing: (1) Participants refused to measure BMD. (2) Pregnancy. (3) History of radiographic contrast agents in the past 7 days. (4) Test weight over 450 pounds (Beyond the measurement range of DXA equipment). (5) History of bilateral hip fractures, bilateral hip replacements, and pins or steel in both hips. (6) Participants with degenerative spine disease include severe scoliosis, stiffening of the spine, previous spinal fusion, laminectomy, and spine fracture. (7) Insufficient scans of the participant's vertebrae and hips to complete the whole scan. (8) Overlapping of body parts during BMD measurement, e.g., overlapping of hands and legs. (9) Other causes were affecting BMD measurements, such as cardiac stenting, morbid obesity producing excessive x-ray noise. A total of 50,463 participants were included in this study initially. After excluded 36,297 participants under 50 years old, 869 participants without BMI data, and 2,387 participants without BMD data, leaving 10,910 participants included in this study finally (Figure 1).

### Definition of Lead Exposure BMI

BMI was calculated as weight (kg) divided by height (m) squared. Participants would not be excluded during the body measurements protocol for medical, safety, or other reasons. Values above the 99th percentile or below the 1st percentile were reviewed for data reasonableness. Data were reviewed for reasonableness based on a combination of height, weight, age, and gender of the participants. Data that were determined to be unrealistic after review will be artificially removed. All data during the BMI measurement would be the original data without any modifications.

### Definition of Outcomes Femoral BMD and Lumbar Spine BMD

The primary outcomes of this study were femoral BMD and lumbar spine BMD. Dual-energy X-ray absorptiometry (DXA) was used to measure the BMD of the participants (19). BMD measurement regions include femoral regions (Total femur, Femoral neck, Trochanter, Intertrochanter, and Ward's triangle) and lumbar spine regions (Total spine, L1, L2, L3, and L4). All measurement data would be subject to data review.

### Definition of Covariates

Demographic variables included Gender (Male, Female), Age, Race (Mexican American, Other Hispanic, Non-Hispanic White, Non-Hispanic Black, and Other Race), Education level (<9th grade, 9–11th grade, High school graduate, Some college or AA degree, and College graduate or above), and Ratio of family income to poverty (0–5). The body covariates included Weight, Standing Height, Arm Circumference, and Waist Circumference. Furthermore, other covariates included Biochemistry (Albumin refrigerated serum, Globulin, Glucose refrigerated serum, Cholesterol refrigerated serum, and Triglycerides refrigerated serum), Moderate work activity, and Smoking-Cigarette Use (Smoked at least 100 cigarettes in life).

### Equipment Information

The Hologic Discovery A: Participants' BMD was examined with the *Hologic Discovery A*. *The Discovery A* used a low level of x-rays, and under standard operating conditions, the entrance dose to the examinee for a whole-body scan is less than one mR1 (a standard x-ray is ~35 mR). Digital weight scale: The participants' weight was weighed using a digital weight scale placed on a flat surface. Portable weight scales: If the participant weighed beyond the scope of the digital weight scale, he or she was weighed on a portable scale. Stadiometer: The participants' height was measured used a stadiometer. Height adjustment ruler: A ruler ~15 cm long was used to correct height if the participant's hairstyle interfered with the measurement or if they were unwilling to remove their shoes.

### Statistical Analysis

Statistical software R (version 3.6.1) and EmpowerStats Software were used to carry out the statistical analysis. Characteristics of the study population were conducted according to BMI subgroup (1) (Underweight, <18.5 kg/m^2^; Normal, 18.5–24.9 kg/m^2^; Overweight, 25–29.9 kg/m^2^; and Obese, ≥30 kg/m^2^). Mean ± standard deviation (SD), and Linear regression model for continuous variables (Age, Ratio of family income to poverty, Albumin refrigerated serum, Globulin, Glucose refrigerated serum, Cholesterol refrigerated serum, Triglycerides refrigerated serum, Weight, Standing Height, Waist Circumference, Arm Circumference, Total femur BMD, Femoral neck BMD, Trochanter BMD, Intertrochanter BMD, Wards triangle BMD, Total spine BMD, L1 BMD, L2 BMD, L3 BMD, L4 BMD. Frequencies (%) and a chi-squared test for categorical variables (Gender, Race, Education level, Moderate work activity, and Smoked at least 100 cigarettes in life). Multivariate linear regression analyses were conducted between the BMI and BMD to calculate the β and *95% confidence interval* (*CI*). Three models were used to construct the multivariate test: Model 1, no covariates were adjusted; Model 2, Gender, Age, and Race were adjusted; Model 3, all covariates were adjusted. All covariates included Gender, Race, Age, Education level, Ratio of family income to poverty, Smoked at least 100 cigarettes in life, Moderate work activity, Albumin refrigerated serum, Globulin, Glucose refrigerated serum, Cholesterol refrigerated serum, Cholesterol Triglycerides, Standing Height, Arm Circumference, Waist Circumference. Whether covariates were adjusted in Model 3, we added them to the basic model or removed them from the full model, changing the BMI β at least 10% or the covariate *P* < 0.1 in the univariate model (Supplementary Table 1). Smoothed curve fittings were performed simultaneously by adjusting the covariates. The association between BMI and BMD was analyzed with a saturated effects analysis model, and the results were expressed as the BMI Turning point (K), effect-β (*95% CI*), and log-likelihood ratio test (*LRT*-test). In the saturated effect analysis model, the covariates were adjusted according to criteria 2 in Supplementary Table 1. Finally, the same multivariate linear regression, smoothed curve fitting, and saturation effects analyses were performed in the gender, age, and race subgroups. *P* < 0.05 was considered statistically significant. Sample weight: The 2-year sample weights were used for all NHANES analyses.

## Results

### Characteristics of the Study Participants

The characteristics of study participants were shown in Table 1. In total, 5,654 male and 5,256 female adults above 50 years old were included. According to the BMI, 163 participants were underweight, 2,781 were normal, 4,185 were overweight, and 3,781 were obese. In each BMI group, the mean for age was 63.236 ± 10.085 years, 63.498 ± 9.887 years, 63.336 ± 9.410 years, and 61.805 ± 8.548 years, respectively. With increased BMI, the femur BMD and spine BMD significantly increased (*P* < 0.00001, respectively). Age, Gender, Race, Weight, Standing Height, Ratio of family income to poverty, Albumin refrigerated serum, Globulin, Glucose refrigerated serum, Cholesterol refrigerated serum, Cholesterol Triglycerides, Waist Circumference, Arm Circumference, Education level, Moderate work activity, and Smoke behavior were also presented in Table 1.

**Table 1:** Characteristics of the study participants.

	**BMI (kg/m** ^**2**^ **) categorical**				***P*-value**
	**Underweight, ≤18.5** **(*N* = 163)**	**Normal, >18.5, ≤25** **(*N* = 2,781)**	**Overweight, >25, ≤30** **(*N* = 4,185)**	**Obese, >30** **(*N* = 3,781)**	
Age (years)	63.236 ± 10.085	63.498 ± 9.887	63.336 ± 9.410	61.805 ± 8.548	<0.00001
Gender (%)					<0.00001
Male	38.683	39.426	55.319	48.435	
Female	61.317	60.574	44.681	51.565	
Race (%)					<0.00001
Mexican American	1.847	3.120	5.605	5.604	
Other Hispanic	0.324	3.089	4.219	3.687	
Non-Hispanic White	72.688	75.851	76.225	76.063	
Non-Hispanic Black	13.944	7.384	7.885	11.474	
Other Race	11.197	10.557	6.066	3.173	
Weight (kg)	48.342 ± 5.896	62.685 ± 9.117	78.265 ± 10.377	97.658 ± 15.646	<0.00001
Standing height (cm)	165.485 ± 8.853	166.370 ± 9.710	168.483 ± 10.309	167.711 ± 10.045	<0.00001
Ratio of family income to poverty	2.387 ± 1.624	3.278 ± 1.598	3.301 ± 1.588	3.270 ± 1.570	<0.00001
Albumin, refrigerated serum (g/L)	41.639 ± 4.164	42.084 ± 3.177	41.958 ± 3.003	40.936 ± 3.032	<0.00001
Globulin (g/L)	29.952 ± 6.323	29.118 ± 5.203	29.176 ± 4.657	29.954 ± 4.718	<0.00001
Glucose, refrigerated serum (mmol/L)	5.371 ± 1.953	5.734 ± 2.248	6.015 ± 2.229	6.423 ± 2.582	<0.00001
Cholesterol, refrigerated serum (mmol/L)	5.135 ± 0.980	5.257 ± 1.058	5.209 ± 1.141	5.072 ± 1.122	<0.00001
Triglycerides, refrigerated serum (mmol/L)	1.092 ± 0.626	1.409 ± 0.873	1.839 ± 1.298	2.089 ± 1.639	<0.00001
Waist Circumference (cm)	72.738 ± 4.940	85.639 ± 7.571	99.079 ± 7.143	114.007 ± 10.537	<0.00001
Arm Circumference (cm)	35.402 ± 2.428	36.510 ± 2.655	37.637 ± 2.767	38.283 ± 2.728	<0.00001
Total femur BMD (g/cm^2^)	0.719 ± 0.130	0.834 ± 0.136	0.929 ± 0.149	0.990 ± 0.150	<0.00001
Femoral neck BMD (g/cm^2^)	0.623 ± 0.116	0.694 ± 0.118	0.763 ± 0.129	0.815 ± 0.140	<0.00001
Trochanter BMD (g/cm^2^)	0.542 ± 0.112	0.632 ± 0.115	0.707 ± 0.129	0.750 ± 0.130	<0.00001
Intertrochanter BMD (g/cm^2^)	0.852 ± 0.159	0.992 ± 0.166	1.104 ± 0.177	1.176 ± 0.179	<0.00001
Wards triangle BMD (g/cm^2^)	0.449 ± 0.115	0.510 ± 0.135	0.563 ± 0.149	0.611 ± 0.166	<0.00001
Total spine BMD (g/cm^2^)	0.829 ± 0.150	0.932 ± 0.148	1.009 ± 0.158	1.059 ± 0.155	<0.00001
L1 BMD (g/cm^2^)	0.758 ± 0.153	0.856 ± 0.159	0.936 ± 0.165	0.999 ± 0.165	<0.00001
L2 BMD (g/cm^2^)	0.837 ± 0.161	0.934 ± 0.163	1.012 ± 0.173	1.066 ± 0.169	<0.00001
L3 BMD (g/cm^2^)	0.862 ± 0.152	0.975 ± 0.166	1.041 ± 0.172	1.092 ± 0.174	<0.00001
L4 BMD (g/cm^2^)	0.877 ± 0.159	0.978 ± 0.164	1.048 ± 0.175	1.097 ± 0.176	<0.00001
Moderate work activity (%)					0.14931
Yes	33.601	38.005	40.236	38.435	
No	66.399	61.882	59.734	61.565	
Education level (%)					<0.00001
<9th grade	9.289	6.346	6.970	5.453	
9–11th grade	15.359	9.484	10.521	10.316	
High school graduate	31.251	23.436	25.371	27.820	
Some college or AA degree	17.784	26.408	27.462	31.744	
College graduate or above	26.317	34.325	29.676	24.667	
Smoked at least 100 cigarettes in life (%)					0.00007
Yes	68.455	48.844	50.008	48.972	
No	31.545	51.156	49.992	51.028	

*Mean ± SD for: Age, Ratio of family income to poverty, Albumin refrigerated serum (g/L), Globulin (g/L), Glucose refrigerated serum (mmol/L), Cholesterol refrigerated serum (mmol/L), Triglycerides refrig serum (mmol/L), Weight (kg), Standing Height (cm), Waist Circumference (cm), Arm Circumference (cm), Total femur BMD (g/cm^2^), Femoral neck BMD (g/cm^2^), Trochanter BMD (g/cm^2^), Intertrochanter BMD (g/cm^2^), Wards triangle BMD (g/cm^2^), Total spine BMD (g/cm^2^), L1 BMD (g/cm^2^), L2 BMD (g/cm^2^), L3 BMD (g/cm^2^), L4 BMD (g/cm^2^). P-value was calculated by weighted linear regression model*.

*Frequencies (%) for Gender, Race, Education level, Moderate work activity, Smoked at least 100 cigarettes in life. The weighted chi-square test calculated the P-value*.

*Weighted by: Full sample 2 year interview weight*.

### The Association Between BMI and BMD

Multivariate linear regression analyses showed BMI was positively associated with Total femur BMD (β = 0.010*, 95% CI* = 0.010, 0.011), Femoral neck BMD (β = 0.014, *95% CI* = 0.012, 0.01), Trochanter BMD (β = 0.013, *95% CI* = 0.012, 0.014), Intertrochanter BMD (β = 0.016, *95% CI* = 0.008, 0.009), Wards triangle BMD (β = 0.013, *95% CI* = 0.011, 0.015), Total spine BMD (β = 0.008, *95% CI* = 0.012, 0.01), L1 BMD (β = 0.011, *95% CI* = 0.009, 0.013), L2 BMD (β = 0.013, *95% CI* = 0.011, 0.015), L3 BMD (β = 0.013, *95% CI* = 0.011, 0.016), and L4 BMD (β = 0.014, *95% CI* = 0.012, 0.017) in the adjusted model (Table 2). When the BMI was categorized for analysis, the higher categorical had a higher BMD in the Total femur in adjusted model (BMI 18.5, 25: β = 0.116, *95% CI* = 0.095, 0.138; BMI 25, 30: β = 0.186, *95% CI* = 0.165, 0.207; BMI ≥ 30: β = 0.247, *95% CI* = 0.226, 0.269). Similarly, Femoral neck, Trochanter, Intertrochanter, Wards triangle, Total spine, L1, L2, L3, and L4 also had a higher BMD in BMI categorical analysis (β*-*Value*, 95% CI*, and *P*-value were showed in Table 2).

**Table 2:** Multiple linear regression analysis of the associations between BMI (kg/m^2^) and BMD (g/cm^2^) in different models.

	**Model**	**BMI (kg/m^**2**^)**	**BMI (kg/m** ^**2**^ **) categorical**				***P* for trend**
			**≤18.5**	**18.5, 25**	**25, 30**	**≥30**	
Total femur BMD	Model 1, β (*95% C*I)	0.012 (0.011, 0.012)	0	0.115 (0.090, 0.139)	0.210 (0.185, 0.234)	0.271 (0.247, 0.296)	*P* < 0.001
	Model 2, β (*95% CI*)	0.011 (0.010, 0.011)	0	0.118 (0.097, 0.139)	0.191 (0.170, 0.211)	0.254 (0.233, 0.275)	
	Model 3, β (*95% CI*)	0.010 (0.010, 0.011)	0	0.116 (0.095, 0.138)	0.186 (0.165, 0.207)	0.247 (0.226, 0.269)	
Femoral neck BMD	Model 1, β (*95% C*I)	0.009 (0.009, 0.010)	0	0.071 (0.049, 0.092)	0.140 (0.118, 0.161)	0.192 (0.170, 0.214)	*P* < 0.001
	Model 2, β (*95% CI*)	0.008 (0.008, 0.009)	0	0.076 (0.056, 0.095)	0.131 (0.112, 0.151)	0.180 (0.161, 0.200)	
	Model 3, β (*95% CI*)	0.014 (0.012, 0.010)	0	0.048 (0.029, 0.060)	0.079 (0.058, 0.100)	0.086 (0.062, 0.109)	
Trochanter BMD	Model 1, β (*95% C*I)	0.009 (0.008, 0.009)	0	0.090 (0.069, 0.111)	0.165 (0.144, 0.186)	0.208 (0.187, 0.229)	*P* < 0.001
	Model 2, β (*95% CI*)	0.008 (0.008, 0.008)	0	0.092 (0.074, 0.111)	0.149 (0.130, 0.167)	0.195 (0.176, 0.213)	
	Model 3, β (*95% CI*)	0.013 (0.012, 0.014)	0	0.065 (0.046, 0.084)	0.097 (0.077, 0.117)	0.103 (0.081, 0.125)	
Intertrochanter BMD	Model 1, β (*95% C*I)	0.014 (0.013, 0.014)	0	0.140 (0.111, 0.169)	0.252 (0.223, 0.281)	0.324 (0.295, 0.353)	*P* < 0.001
	Model 2, β (*95% CI*)	0.013 (0.012, 0.013)	0	0.143 (0.118, 0.169)	0.230 (0.204, 0.255)	0.304 (0.279, 0.329)	
	Model 3, β (*95% CI*)	0.016 (0.014, 0.018)	0	0.100 (0.074, 0.126)	0.145 (0.118, 0.173)	0.155 (0.125, 0.186)	
Wards triangle BMD	Model 1, β (*95% C*I)	0.008 (0.007, 0.008)	0	0.061 (0.036, 0.086)	0.114 (0.089, 0.140)	0.163 (0.137, 0.188	*P* < 0.001
	Model 2, β (*95% CI*)	0.007 (0.006, 0.007)	0	0.066 (0.043, 0.090)	0.114 (0.091, 0.137)	0.153 (0.130, 0.177)	
	Model 3, β (*95% CI*)	0.013 (0.011, 0.015)	0	0.047 (0.022, 0.071)	0.078 (0.052, 0.103)	0.085 (0.057, 0.114)	
Total spine BMD	Model 1, β (*95% C*I)	0.010 (0.009, 0.010)	0	0.103 (0.073, 0.133)	0.180 (0.150, 0.210)	0.230 (0.200, 0.260)	*P* < 0.001
	Model 2, β (*95% CI*)	0.009 (0.008, 0.009)	0	0.107 (0.078, 0.135)	0.169 (0.141, 0.197)	0.220 (0.192, 0.248)	
	Model 3, β (*95% CI*)	0.008 (0.008, 0.009)	0	0.112 (0.083, 0.141)	0.171 (0.142, 0.200)	0.218 (0.189, 0.247)	
L1BMD	Model 1, β (*95% C*I)	0.010 (0.010, 0.011)	0	0.099 (0.070, 0.127)	0.178 (0.150, 0.206)	0.241 (0.213, 0.269)	*P* < 0.001
	Model 2, β (*95% CI*)	0.010 (0.009, 0.010)	0	0.100 (0.074, 0.126)	0.159 (0.134, 0.185)	0.227 (0.201, 0.252)	
	Model 3, β (*95% CI*)	0.011 (0.009, 0.013)	0	0.073 (0.047, 0.100)	0.107 (0.079, 0.136)	0.135 (0.103, 0.167)	
L2 BMD	Model 1, β (*95% C*I)	0.009 (0.009, 0.010)	0	0.098 (0.068, 0.127)	0.176 (0.146, 0.205)	0.229 (0.200, 0.259)	*P* < 0.001
	Model 2, β (*95% CI*)	0.009 (0.008, 0.009)	0	0.101 (0.073, 0.128)	0.160 (0.133, 0.187)	0.215 (0.187, 0.242)	
	Model 3, β (*95% CI*)	0.013 (0.011, 0.015)	0	0.081 (0.053, 0.110)	0.121 (0.090, 0.151)	0.143 (0.108, 0.177)	
L3 BMD	Model 1, β (*95% C*I)	0.009 (0.008, 0.009)	0	0.113 (0.082, 0.143)	0.178 (0.148, 0.209)	0.229 (0.199, 0.260)	*P* < 0.001
	Model 2, β (*95% CI*)	0.008 (0.008, 0.009)	0	0.117 (0.087, 0.146)	0.168 (0.139, 0.197)	0.222 (0.193, 0.251)	
	Model 3, β (*95% CI*)	0.013 (0.011, 0.016)	0	0.101 (0.070, 0.131)	0.136 (0.104, 0.169)	0.161 (0.125, 0.198)	
L4 BMD	Model 1, β (*95% C*I)	0.009 (0.008, 0.009)	0	0.101 (0.069, 0.133)	0.171 (0.140, 0.203)	0.220 (0.188, 0.252)	*P* < 0.001
	Model 2, β (*95% CI*)	0.008 (0.008, 0.009)	0	0.107 (0.077, 0.138)	0.164 (0.133, 0.194)	0.217 (0.187, 0.248)	
	Model 3, β (*95% CI*)	0.014 (0.012, 0.017)	0	0.089 (0.058, 0.121)	0.127 (0.094, 0.161)	0.146 (0.108, 0.184)	

*Results: β (95% CI)*.

*Outcome: Total femur BMD, Femoral neck BMD, Trochanter BMD, Intertrochanter BMD, Wards triangle BMD, Total spine BMD, L1 BMD, L2 BMD, L3 BMD, and L4 BMD*.

*Exposure: BMI (kg/m^2^)*.

*Model 1 adjusts for None*.

*Model 2 adjusted for Gender, Age, Race*.

*Model 3 adjusted for: Gender; Race; Gender, Race, Age, Education level, Ratio of family income to poverty, Smoked at least 100 cigarettes in life, Moderate work activity, Albumin refrigerated serum, Globulin, Glucose refrigerated serum, Cholesterol refrigerated serum, Cholesterol Triglycerides, Standing Height, Arm Circumference, Waist Circumference*.

*Weighted by: Full sample 2 year interview weight*.

**Figure 2:**
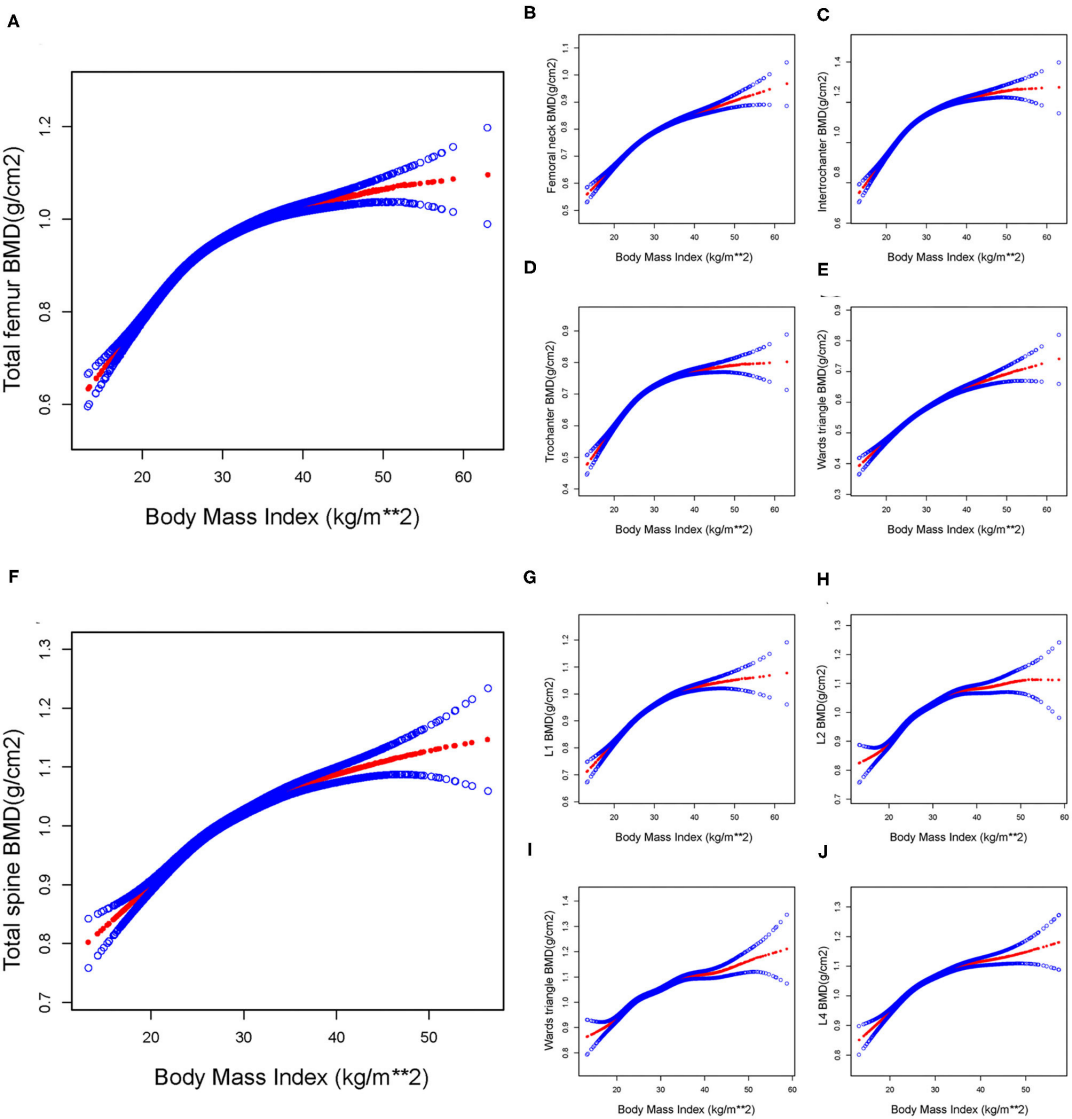
The association between BMI and BMD in total participants. By saturation effect analysis, saturation values were found in all subgroups (Log-likelihood ratio test, *P* < 0.00*1*, respectively). **(A)** Total femur group, BMI saturation value = 26.13 (kg/m^2^). **(B)** Femoral neck group, BMI saturation value = 26.59 (kg/m^2^). **(C)** Trochanter group, BMI saturation value = 26.44 (kg/m^2^). **(D)** Intertrochanter group, BMI saturation value = 26.06 (kg/m^2^). **(E)** Ward triangle group, BMI saturation value = 29.60 (kg/m^2^). **(F)** Total spine group, BMI saturation value = 26.82 (kg/m^2^). **(G)** L1 group, BMI saturation value = 31.90 (kg/m^2^). **(H)** L2 group, BMI saturation value = 30.89 (kg/m^2^). **(I)** L3 group, BMI saturation value = 25.60 (kg/m^2^). **(J)** L4 group, BMI saturation value = 25.60 (kg/m^2^). The red line represents the β*-*value, and the blue line represents the *95% CI*. Adjust for: Gender, Race, Age, Education level, Ratio of family income to poverty, Smoked at least 100 cigarettes in life, Moderate work activity, Albumin refrigerated serum, Globulin, Glucose refrigerated serum, Cholesterol refrigerated serum, Cholesterol Triglycerides, Standing Height, Arm Circumference, Waist Circumference.

When a smoothing curve fitting was conducted in the adjusted model (Figure 2), we found a saturation effect value between BMI and BMD. We further used the saturation effect analysis model to investigate the BMI turning point and found that the saturation effect value was 26.13 (kg/m^2^) in the Total femur BMD, 26.59 (kg/m^2^) in the Femoral neck BMD, 26.44 (kg/m^2^) in the Trochanter BMD, 26.06 (kg/m^2^) in the Intertrochanter BMD, 29.60 (kg/m^2^) in the Wards triangle BMD, 26.82 (kg/m^2^) in the Total spine BMD, 31.90 (kg/m^2^) in the L1 BMD, 30.89 (kg/m^2^) in the L2 BMD, 25.60 (kg/m^2^) in the L3 BMD, and 25.60 (kg/m^2^) in the L4 BMD (Table 3). When BMI was <26.13 (kg/m^2^), the total femur BMD increased by 0.023 (*95% CI* = 0.022, 0.025) g/cm^2^ for each unit increase in BMI. However, when BMI exceeded 26.13 (kg/m^2^), the total femur BMD increased by only 0.007 (*95% CI* = 0.007, 0.008) g/cm^2^ for each unit increase in BMI. Similarly, when BMI was less than the turning point, BMD of the Femoral neck, Trochanter, Intertrochanter, Wards triangle, Total spine, L1, L2, L3, and L4 increased by 0.016 (*95% CI* = 0.015, 0.017), 0.018 (*95% CI* = 0.017, 0.020), 0.028 (*95% CI* = 0.026, 0.030), 0.013 (*95% CI* = 0.011, 0.014), 0.018 (*95% CI* = 0.016, 0.020), 0.015 (*95% CI* = 0.014, 0.016), 0.015 (*95% CI* = 0.014, 0.016), 0.018 (*95% CI* = 0.016, 0.020), and 0.018 (*95% CI* = 0.016, 0.020) g/cm^2^, respectively, for each unit increase in BMI. However, when BMI exceeds the turning point, BMD at the sites mentioned above increases very slowly (Table 3).

**Table 3:** Saturation effect analysis of BMI (kg/m^2^) on BMD (g/cm^2^) in whole participants.

**Outcome**	**Model: Saturation effect analysis**				***LRT*-test**
	**BMI turning point (K), kg/m^**2**^**	**<K, effect 1**	**>K, effect 2**	**Effect 2 – 1**	
Total femur BMD	26.13	0.023 (0.022, 0.025)	0.007 (0.007, 0.008)	−0.016 (−0.018, −0.014)	<0.001
Femoral neck BMD	26.59	0.016 (0.015, 0.017)	0.007 (0.006, 0.007)	−0.009 (−0.011, −0.008)	<0.001
Trochanter BMD	26.44	0.018 (0.017, 0.020)	0.005 (0.004, 0.006)	−0.013 (−0.015, −0.012)	<0.001
Intertrochanter BMD	26.06	0.028 (0.026, 0.030)	0.008 (0.007, 0.009)	−0.020 (−0.022, −0.017)	<0.001
Wards triangle BMD	29.60	0.013 (0.011, 0.014)	0.006 (0.005, 0.007)	−0.006 (−0.008, −0.005)	<0.001
Total spine BMD	26.82	0.018 (0.016, 0.020)	0.006 (0.005, 0.007)	−0.012 (−0.015, −0.010)	<0.001
L1 BMD	31.90	0.015 (0.014, 0.016)	0.004 (0.003, 0.005)	−0.012 (−0.014, −0.010)	<0.001
L2 BMD	30.89	0.015 (0.014, 0.016)	0.002 (0.001, 0.003)	−0.013 (−0.015, −0.011)	<0.001
L3 BMD	25.60	0.018 (0.016, 0.020)	0.006 (0.005, 0.007)	−0.012 (−0.014, −0.009)	<0.001
L4 BMD	25.60	0.018 (0.016, 0.020)	0.006 (0.005, 0.006)	−0.012 (−0.015, −0.010)	<0.001

*Results in the table: β (95% CI)*.

*Outcome: Total femur BMD, Femoral neck BMD, Femoral neck BMD, Trochanter BMD, Intertrochanter BMD, Wards triangle BMD, Total spine BMD, L1 BMD, L2 BMD, L3 BMD, L4 BMD*.

*Exposure: BMI (kg/m^2^)*.

*Adjust for: Gender, Race, Age, Education level, Ratio of family income to poverty, Smoked at least 100 cigarettes in life, Moderate work activity, Albumin refrigerated serum, Globulin, Glucose refrigerated serum, Cholesterol refrigerated serum, Cholesterol Triglycerides, Standing Height, Arm Circumference, Waist Circumference*.

*LRT-test: Log-likelihood ratio test*.

### Subgroup Analysis

The subgroup analysis conducted by gender was shown in Supplementary Table 2 (Male) and Supplementary Table 3 (Female). After adjusting the covariates, the smoothing curve fitting showed a saturation effect value between BMI and BMD in male participants (Supplementary Figure 1). After adjusting for covariates and using a saturated effects model, we found that the BMI saturation value was 26.25 (kg/m^2^) in total femur BMD and 26.84 (kg/m^2^) in the total spine in males participants. This was consistent with the results obtained for the whole participants. However, saturation effects were not evident in the female participants, and saturation values for each femur and lumbar spine region varied widely (Supplementary Figure 2).

The subgroup analysis conducted by age was shown in Supplementary Table 4. After adjusting the covariates, the smoothing curve fitting also showed a saturation effect value. The saturation effect model showed that in the participants aged 60–70 years, the saturation values of BMI in the Femoral neck, Ward triangle, Total spine, and L1–L4 reached 32 (kg/m^2^), and the results in other age groups were similar in the whole participants (Supplementary Figure 3).

The subgroup analysis conducted by race was shown in Supplementary Table 5. The results showed that the non-Hispanic population had a higher BMI saturation value at the lumbar spine region than at around 30 (kg/m^2^), while others had a BMI saturation value at around 24 (kg/m^2^). Mexican Americans and other Hispanics had higher BMI saturation values than other races at several sites in the femoral region, reaching around 35 (kg/m^2^) in some areas (Supplementary Figure 4).

## Discussion

In the present study, a BMI saturation value (around 26 kg/m^2^) was founded in the femur BMD and spine BMD in all participants over 50 years old. A positive association was found between BMI and BMD at BMI level <26 (kg/m^2^) and a minimal increase in BMI level above 26 (kg/m^2^), this being important for maintaining optimal BMD.

A positive association was found between BMI and BMD. When BMI was <26 (kg/m^2^), for each unit increased in BMI, the BMD of the femur and spine increased by 0.023 (g/cm^2^) and 0.018 (g/cm^2^), which was consistent with the previous studies (20, 21). A cross-sectional study in a Polish population also showed a positive association between BMI and BMD (22). Another study by Morin et al. indicated that low BMI predicted the future occurrence of osteoporosis and increased fracture risk (23). In the middle-aged and elderly female population, maintaining obesity could prevent the onset of postmenopausal bone loss (24). Similarly, a 10.5-year prospective cohort study suggested that obesity may be associated with the delayed bone loss (25). There were several possible mechanisms for a positive association between BMI and BMD as follows: (1) The increased static mechanical compliance produced by excessive fat accumulation was one of the possible mechanisms. Excessive fat accumulation and high body weight could impose greater static mechanical loads on the bones, and bone tissue produced a series of changes when it felt the mechanical forces exerted by the body (26, 27). (2) More body fat in patients with high BMI was accompanied by an increase in various hormones, such as estrogen (28), insulin (29), and leptin (30), which had a beneficial effect on BMD by inhibiting bone resorption (31) and bone remodeling (32, 33). (3) Androgens in adipose tissue were converted to estrogen, which would increase bone mass (34). (4) Studies also reported the effect of some genes (35) on BMD, such as the mutation of the Pro10 allele in tumor necrosis factor-β1 (TGF-β1), which is more frequent in obese patients (36).

Nevertheless, when BMI exceeded a specific value of 26 (kg/m^2^), the BMD of the femur and spine increased with each unit of BMI by only 0.007 (g/cm^2^) and 0.006 (g/cm^2^). The mechanisms of keeping the BMI around 26 to have the most optimal BMD had not been fully explained. A review of the literature allowed us to draw some possible clues. (1) Genetic determinism. Bone growth trajectories (37) and peak bone mass (38) were determined early in life, which might be one of the possible explanations that BMD no longer increased after a limited increase in value in adults. Pocock et al. (39) explored genetic effects on BMD in 38 identical and 27 dizygotic twins and found that genetics determines adult bone mass. Genetic influences explained 75% of BMD variance, regardless of whether the twin was male (40) or female (41). Meanwhile, other acquired environmental factors, such as increased BMI (21), calcium intake (42), estrogen intake (43), and physical exercise (44), have a limit to the increase in BMD in adults. (2) Multi-factors co-leading. Researches had shown a specific bone-adipose axis (45) between adipose and bone tissues within the body, connected by a variety of bioactive molecules and maintained bone homeostasis, which might be another possible mechanism for the presence of BMI saturation effects. Available studies clarified that bone and adipocytes originated from a common stem cell precursor and were competitive, with excess fat gain leading to bone loss (46, 47). Several experiments in animal models induced by high-fat diets had confirmed that BMD in obese animals decreased with increasing obesity (48–50). The PPAR-γ (Peroxisome Proliferator-Activated Receptor-γ) pathway (46) was involved in adipose and bone differentiation in obesity animal models *in vivo*. Activation of PPAR-γ (51) stimulates adipogenesis and bone loss, while inhibition of PPAR-γ prevents (52) bone loss. Other studies had shown that adipose was an endocrine organ that secreted inflammatory cells such as interleukin-1 (IL-1) and tumor necrosis factor-α (TNF-α) (53). The above cytokines inhibit BMD via the OPG/RANKL/RANK pathway (54). We hypothesized that the multiple factors mentioned above combined to cause an increase in BMI to a certain range without a significant increase in BMD, but direct experimental evidence was insufficient, so more advanced studies were still needed.

Our results showed that although the BMI saturation values were similar in the total femur (BMI saturation value, 26.13 kg/m^2^) and total spine (BMI saturation value, 26.82 kg/m^2^), they were significantly higher in the L1 (BMI saturation value, 31.09 kg/m^2^) and L2 (BMI saturation value, 30.89 kg/m^2^) than in the other sites, showed site-specificity. This might be related to the increased spondylarthrosis in the elderly, which led to reactive changes and increasing BMD in the lumbar (55). Unfortunately, we could not extract osteoarthritis-related data for a related study because of limitations in the database itself. Subgroup analysis by gender showed that the BMI saturation values for male participants' femur and spine were concentrated around 26 kg/m^2^. However, the BMI saturation values for female participants varied greatly by each site, which might be linked to the different levels of sex hormones in males and females (56). Age-subgrouped analysis showed that people aged 60–70 years had higher BMI saturation values for the spine than other age groups. A 10-year survey of bone loss rates in the elderly population from Japan suggested that this was related to the prevalence of spine osteophytes in this age group (57). In addition, race subgroup analysis revealed significant differences in BMI saturation values across races, suggesting that different genetic backgrounds and ancestry might be associated with this phenomenon (58).

Indeed, excessive BMI was detrimental to the elderly population. For one, high BMI brought a range of bone-related diseases, such as increased bone fragility (59) and increased fracture risk (60). On the other hand, obesity could lead to a variety of chronic diseases and complications, such as cardiovascular disease (61), type 2 diabetes (62), gallbladder disease (63), and fatty liver (64). More seriously, excessive obesity might be associated with increased cancer risk (65) and cancer-related mortality (66). Therefore, we believed that keeping BMI at a reasonable value (around 26 kg/m^2^) would maintain optimal BMD and reduce the risk of other obesity-related diseases and complications.

There were several limitations in this study. Firstly, this was a cross-sectional study, and we could only conclude whether there was an association between BMI and BMD, not a direct cause-and-effect correlation. Secondly, due to limitations in the database itself, we could not identify the medication-taking participants, menstrual and menopausal (female participants), and osteoarthritis participants, so our conclusions need to be interpreted with caution. Thirdly, we performed covariates detection to control confounders, but there might still be unpredictable covariates, such as medicine use, menstrual status (female), body fat percentage, etc. Last but not least, to the best of our knowledge, the present study was conducted in a US population, and the findings should be cautiously extended to other populations.

## Conclusion

In the present study, we used the multivariate linear regression models, smoothing curve fitting, and saturation effects analysis models to investigate the association between BMI and BMD of people over 50 years old in the US. We found not only a simple linear positive association between BMI and BMD, but a saturation value existed, and this saturation value persisted during site-specific analysis, sex, age, and race subgroup analysis. This work indicated that keeping BMI at a reasonable value (around 26 kg/m^2^) will provide the most significant benefit to older adults in maintaining optimal BMD and reducing other obesity-related diseases.

## Data Availability Statement

The original contributions presented in the study are included in the article/Supplementary Material, further inquiries can be directed to the corresponding author/s.

## Ethics Statement

The studies involving human participants were reviewed and approved by NCHS IRB/ERB Protocol Number: Protocol #2005-06, Continuation of Protocol #2005-06, Continuation of Protocol #2011-17, Protocol #2018-01. The patients/participants provided their written informed consent to participate in this study.

## Author Contributions

MM and ZF conceived the study, data curation, data analysis, and draft writing. XL and GJ completed images and tables preparation. BG conceived the study and writing Instruction. YX conceived the study, funding acquisition, and writing-review editing. All the authors participated in critical revision of the manuscript, contributed to the article, and approved the submitted version.

## Funding

This work was supported by the National Natural Science Foundation of China (81874017, 81960403, and 82060405); Natural Science Foundation of Gansu Province of China (20JR5RA320); and Cuiying Scientific and Technological Innovation Program of Lanzhou University Second Hospital (CY2017-ZD02).

## Conflict of Interest

The authors declare that the research was conducted in the absence of any commercial or financial relationships that could be construed as a potential conflict of interest.

## Publisher's Note

All claims expressed in this article are solely those of the authors and do not necessarily represent those of their affiliated organizations, or those of the publisher, the editors and the reviewers. Any product that may be evaluated in this article, or claim that may be made by its manufacturer, is not guaranteed or endorsed by the publisher.
