# Supplementary material for: The Saturation Effect of Body Mass Index on Bone Mineral Density for People Over 50 Years Old: A Cross-Sectional Study of the US Population
Source: Front Nutr. 2021 Oct 15;8:763677. doi: 10.3389/fnut.2021.763677 (PMC8554069; doi:10.3389/fnut.2021.763677)
Supplement: Supplementary file 1 [file Data_Sheet_1.DOCX]

Supplementary Material

## Supplementary Figures


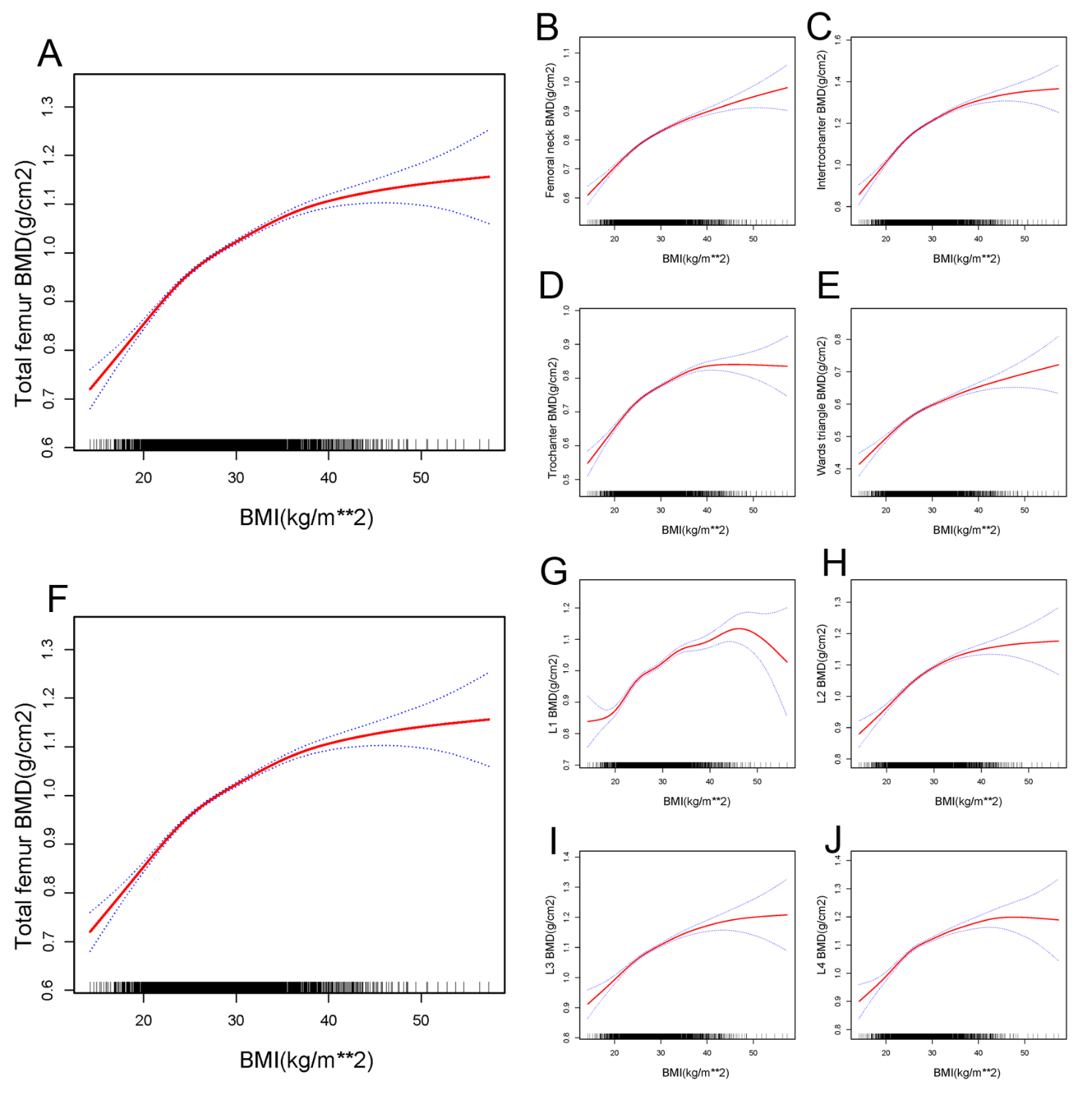


**Supplementary Figure 1.** The association between BMI and BMD in male participants.


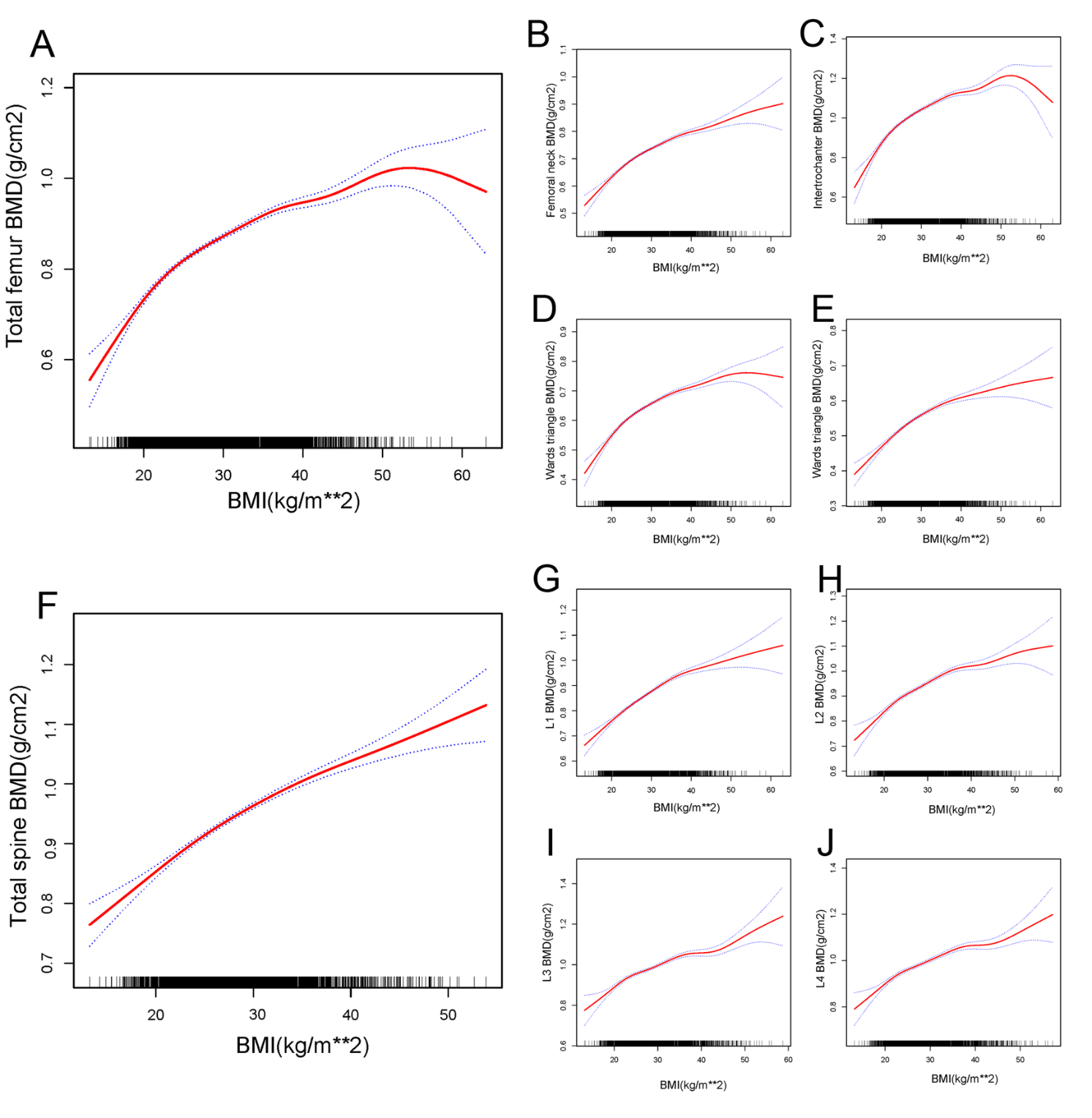


**Supplementary Figure 2.** The association between BMI and BMD in female participants.


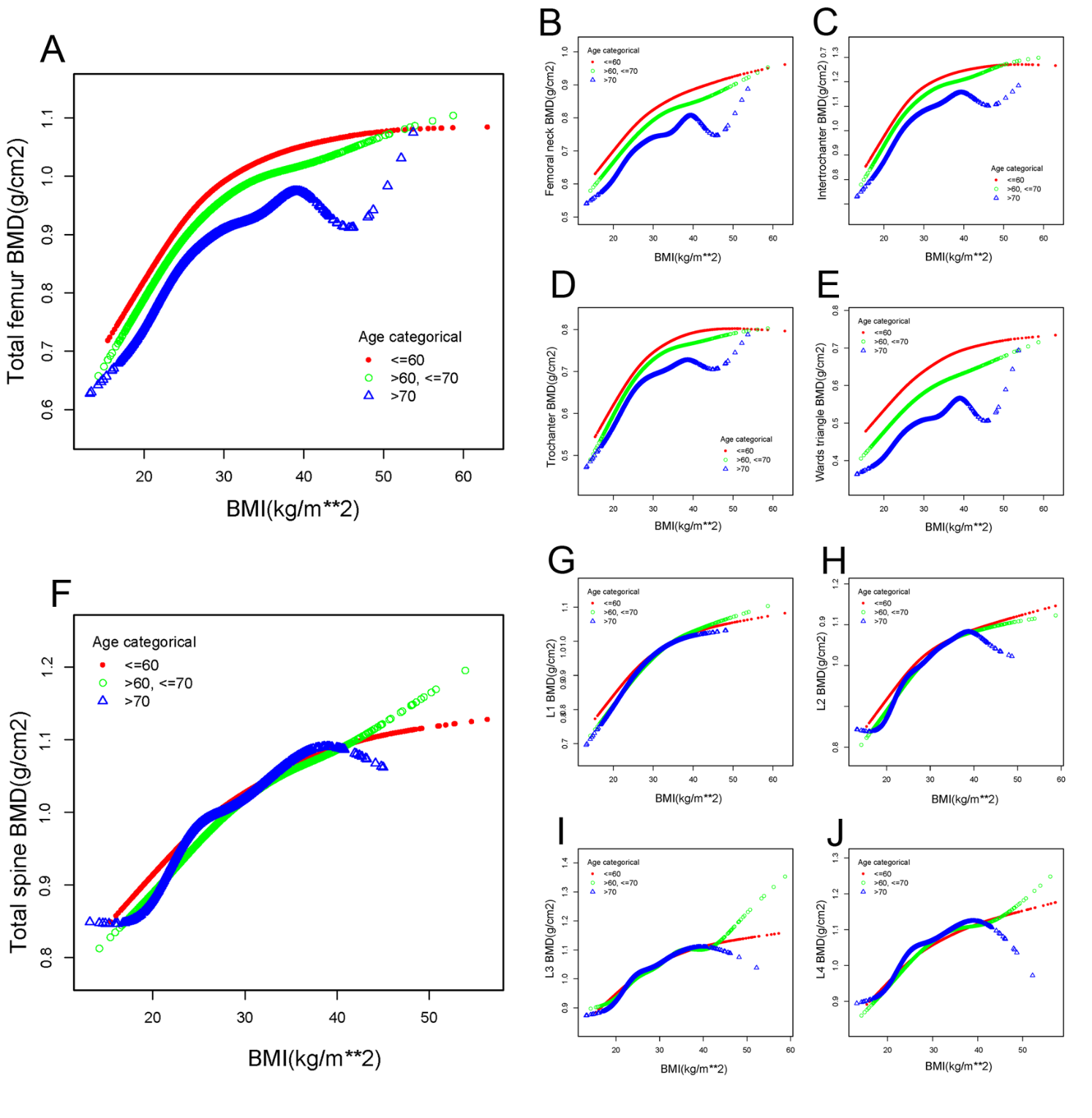


**Supplementary Figure 3.** The association between BMI and BMD in age subgroup.


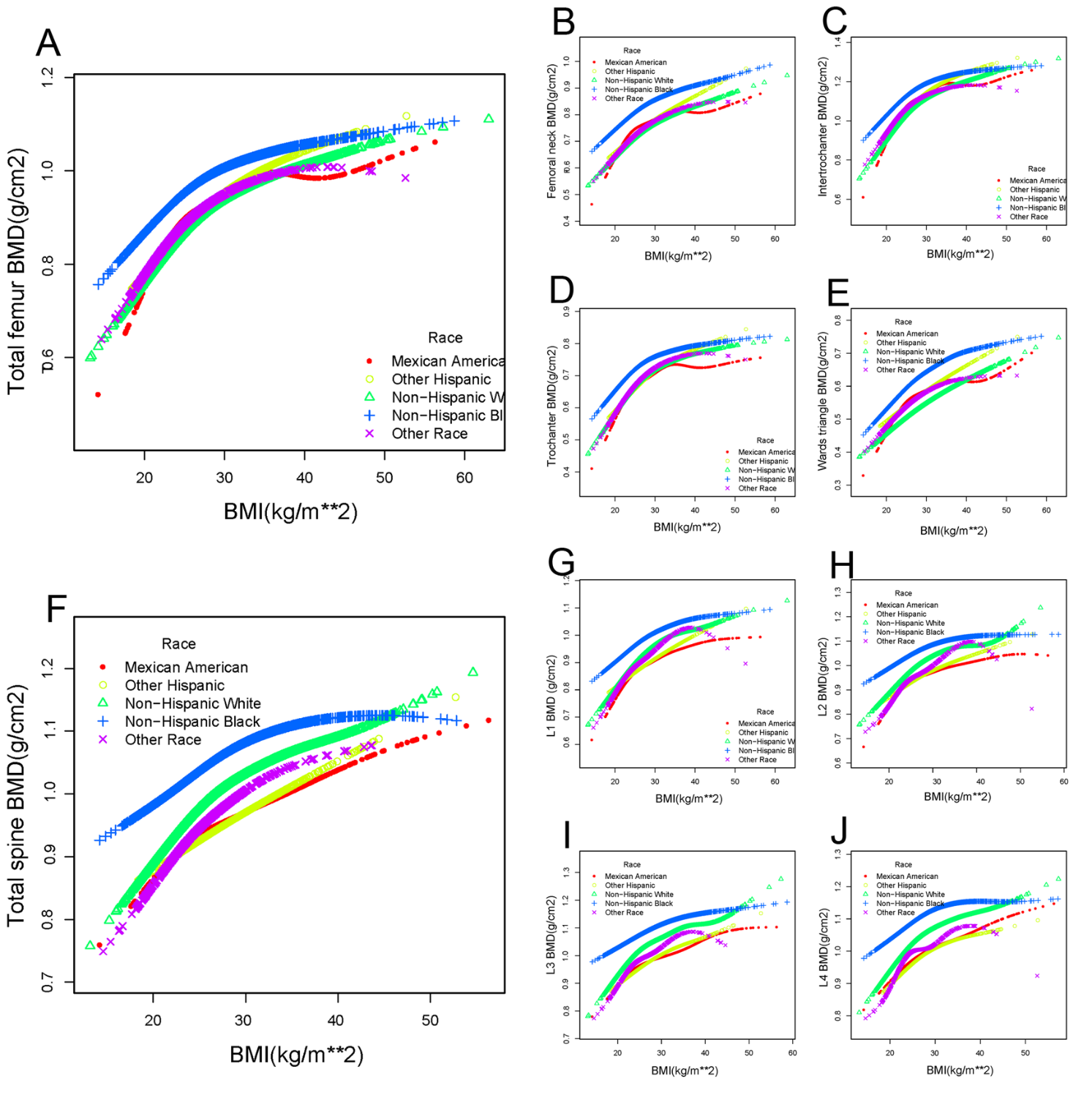


**Supplementary Figure 4.** The association between BMI and BMD in race subgroup.

## Supplementary Tables

**Supplementary Table 1.** Selection of covariates

| Y | X | Selected covariates（Criteria 1） | Selected covariates（Criteria 2） |
| --- | --- | --- | --- |
| Total femur BMD(g/cm^2^) | Body Mass Index (kg/m^2^) | Age(Years)  Standing Height (cm) | Gender  Race  Age  Education level  Ratio of family income to poverty  Moderate work activity  Smoked at least 100 cigarettes in life  Albumin refrigerated serum  Globulin  Glucose refrigerated serum  Cholesterol refrigerated serum  Triglycerides refrigerated serum  Standing Height  Arm Circumference  Waist Circumference |
| Femoral neck BMD(g/cm^2^) | Body Mass Index (kg/m^2^) | Age(Years)  Standing Height (cm) | Gender  Race  Age  Education level  Ratio of family income to poverty  Moderate work activity  Smoked at least 100 cigarettes in life  Albumin refrigerated serum  Globulin  Glucose refrigerated serum  Cholesterol refrigerated serum  Triglycerides refrigerated serum  Standing Height  Arm Circumference  Waist Circumference |
| Trochanter BMD(g/cm^2^) | Body Mass Index (kg/m^2^) | Standing Height (cm) | Gender  Race  Age  Education level  Ratio of family income to poverty  Moderate work activity  Smoked at least 100 cigarettes in life  Albumin refrigerated serum  Globulin  Glucose refrigerated serum  Cholesterol refrigerated serum  Triglycerides refrigerated serum  Standing Height  Arm Circumference  Waist Circumference |
| Intertrochanter BMD(g/cm^2^) | Body Mass Index (kg/m^2^) | Age(Years)  Standing Height (cm) | Gender  Race  Age  Education level  Ratio of family income to poverty  Moderate work activity  Smoked at least 100 cigarettes in life  Albumin refrigerated serum  Globulin  Glucose refrigerated serum  Cholesterol refrigerated serum  Triglycerides refrigerated serum  Standing Height  Arm Circumference  Waist Circumference |
| Wards triangle BMD(g/cm^2^) | Body Mass Index (kg/m^2^) | Age(Years)  Standing Height (cm) | Gender  Race  Age  Education level  Ratio of family income to poverty  Moderate work activity  Smoked at least 100 cigarettes in life  Albumin refrigerated serum  Globulin  Glucose refrigerated serum  Cholesterol refrigerated serum  Triglycerides refrigerated serum  Standing Height  Arm Circumference  Waist Circumference |
| Total spine BMD(g/cm^2^) | Body Mass Index (kg/m^2^) | Standing Height (cm) | Gender  Race  Age  Education level  Ratio of family income to poverty  Moderate work activity  Smoked at least 100 cigarettes in life  Albumin refrigerated serum  Globulin  Glucose refrigerated serum  Cholesterol refrigerated serum  Triglycerides refrigerated serum  Standing Height  Arm Circumference  Waist Circumference |
| L1 BMD(g/cm^2^) | Body Mass Index (kg/m^2^) | Standing Height (cm) | Gender  Race  Age  Education level  Ratio of family income to poverty  Moderate work activity  Smoked at least 100 cigarettes in life  Albumin refrigerated serum  Globulin  Glucose refrigerated serum  Cholesterol refrigerated serum  Triglycerides refrigerated serum  Standing Height  Arm Circumference  Waist Circumference |
| L2 BMD(g/cm^2^) | Body Mass Index (kg/m^2^) | Standing Height (cm) | Gender  Race  Age  Education level  Ratio of family income to poverty  Moderate work activity  Smoked at least 100 cigarettes in life  Albumin refrigerated serum  Globulin  Glucose refrigerated serum  Cholesterol refrigerated serum  Triglycerides refrigerated serum  Standing Height  Arm Circumference  Waist Circumference |
| L3 BMD(g/cm^2^) | Body Mass Index (kg/m^2^) | Standing Height (cm) | Gender  Race  Age  Education level  Ratio of family income to poverty  Moderate work activity  Smoked at least 100 cigarettes in life  Albumin refrigerated serum  Globulin  Glucose refrigerated serum  Cholesterol refrigerated serum  Triglycerides refrigerated serum  Standing Height  Arm Circumference  Waist Circumference |
| L4 BMD(g/cm^2^) | Body Mass Index (kg/m^2^) | Age(Years)  Standing Height (cm) | Gender  Race  Age  Education level  Ratio of family income to poverty  Moderate work activity  Smoked at least 100 cigarettes in life  Albumin refrigerated serum  Globulin  Glucose refrigerated serum  Cholesterol refrigerated serum  Triglycerides refrigerated serum  Standing Height  Arm Circumference  Waist Circumference |

Criteria 1：add the covariate to the basic model or remove it from the full model, change X coefficient >10%
Criteria 2：criteria 1 or the covariate P<0.1 in univariate model vs Y

**Supplementary Table 2.** Saturation effect analysis of BMI (kg/m^2^) on BMD (g/cm^2^) in male participants.

| Outcome | Model: Saturation effect analysis | | | | *LRT* test |
| --- | --- | --- | --- | --- | --- |
|  | BMI Turning point(K)  kg/m^2^ | < K, effect 1 | > K , effect 2 | effect 2 - 1 |  |
| Total femur BMD | 26.25 | 0.023 (0.020, 0.025) <0.0001 | 0.009 (0.008, 0.010) <0.0001 | -0.014 (-0.016, -0.011) <0.0001 | <0.001 |
| Femoral neck BMD | 26.40 | 0.015 (0.013, 0.017) <0.0001 | 0.008 (0.007, 0.008) <0.0001 | -0.008 (-0.010, -0.005) <0.0001 | <0.001 |
| Trochanter BMD | 26.40 | 0.017 (0.015, 0.019) <0.0001 | 0.006 (0.005, 0.007) <0.0001 | -0.011 (-0.013, -0.008) <0.0001 | <0.001 |
| Intertrochanter BMD | 26.13 | 0.028 (0.025, 0.030) <0.0001 | 0.010 (0.009, 0.011) <0.0001 | -0.017 (-0.021, -0.014) <0.0001 | <0.001 |
| Wards triangle BMD | 26.10 | 0.013 (0.011, 0.016) <0.0001 | 0.006 (0.005, 0.007) <0.0001 | -0.007 (-0.010, -0.004) <0.0001 | <0.001 |
| Total spine BMD | 26.84 | 0.018 (0.015, 0.020) <0.0001 | 0.005 (0.004, 0.007) <0.0001 | -0.012 (-0.016, -0.009) <0.0001 | <0.001 |
| L1 BMD | 26.24 | 0.020 (0.017, 0.023) <0.0001 | 0.007 (0.006, 0.008) <0.0001 | -0.013 (-0.016, -0.010) <0.0001 | <0.001 |
| L2 BMD | 29.32 | 0.015 (0.014, 0.017) <0.0001 | 0.004 (0.002, 0.006) <0.0001 | -0.012 (-0.015, -0.009) <0.0001 | <0.001 |
| L3 BMD | 29.90 | 0.013 (0.011, 0.015) <0.0001 | 0.004 (0.002, 0.006) 0.0006 | -0.010 (-0.013, -0.006) <0.0001 | <0.001 |
| L4 BMD | 26.13 | 0.018 (0.015, 0.021) <0.0001 | 0.006 (0.005, 0.008) <0.0001 | -0.012 (-0.016, -0.008) <0.0001 | <0.001 |

Results in the table: *β* (*95% CI*) *P*-value

Outcome: Total femur BMD, Femoral neck BMD, Femoral neck BMD, Trochanter BMD, Intertrochanter BMD, Wards triangle BMD, Total spine BMD, L1 BMD, L2 BMD, L3 BMD, L4 BMD.

Exposure: BMI (kg/m^2^)

Adjust for: Gender, Race, Age, Education level, Ratio of family income to poverty, Smoked at least 100 cigarettes in life, Moderate work activity, Albumin refrigerated serum, Globulin, Glucose refrigerated serum, Cholesterol refrigerated serum, Cholesterol Triglycerides, Standing Height, Arm Circumference, Waist Circumference.

*LRT* test: Log-likelihood ratio test.

**Supplementary Table 3.** Saturation effect analysis of BMI (kg/m^2^) on BMD (g/cm^2^) in female participants.

| Outcome | Model: Saturation effect analysis | | | | *LRT* test |
| --- | --- | --- | --- | --- | --- |
|  | BMI Turning point(K),  kg/m^2^ | < K, effect 1 | > K , effect 2 | effect 2 - 1 |  |
| Total femur BMD | 22.35 | 0.024 (0.020, 0.028) <0.0001 | 0.010 (0.009, 0.010) <0.0001 | -0.014 (-0.019, -0.010) <0.0001 | <0.001 |
| Femoral neck BMD | 36.66 | 0.010 (0.009, 0.010) <0.0001 | 0.005 (0.003, 0.007) <0.0001 | -0.005 (-0.007, -0.003) <0.0001 | <0.001 |
| Trochanter BMD | 22.48 | 0.019 (0.016, 0.023) <0.0001 | 0.007 (0.007, 0.008) <0.0001 | -0.012 (-0.015, -0.008) <0.0001 | <0.001 |
| Intertrochanter BMD | 22.48 | 0.029 (0.024, 0.034) <0.0001 | 0.011 (0.011, 0.012) <0.0001 | -0.018 (-0.023, -0.013) <0.0001 | <0.001 |
| Wards triangle BMD | 37.00 | 0.009 (0.008, 0.010) <0.0001 | 0.002 (-0.001, 0.005) 0.1169 | -0.007 (-0.010, -0.004) <0.0001 | <0.001 |
| Total spine BMD | 21.91 | 0.020 (0.014, 0.026) <0.0001 | 0.009 (0.008, 0.009) <0.0001 | -0.012 (-0.018, -0.005) 0.0006 | <0.001 |
| L1 BMD | 35.80 | 0.011 (0.010, 0.012) <0.0001 | 0.005 (0.002, 0.007) 0.0004 | -0.007 (-0.010, -0.004) <0.0001 | <0.001 |
| L2 BMD | 34.00 | 0.011 (0.010, 0.012) <0.0001 | 0.005 (0.003, 0.007) <0.0001 | -0.006 (-0.009, -0.003) <0.0001 | <0.001 |
| L3 BMD | 21.00 | 0.027 (0.018, 0.036) <0.0001 | 0.008 (0.007, 0.009) <0.0001 | -0.019 (-0.028, -0.010) <0.0001 | <0.001 |
| L4 BMD | 20.37 | 0.031 (0.019, 0.043) <0.0001 | 0.008 (0.007, 0.009) <0.0001 | -0.024 (-0.036, -0.011) 0.0002 | <0.001 |

Results in the table: *β* (*95% CI*) *P*-value

Outcome: Total femur BMD, Femoral neck BMD, Femoral neck BMD, Trochanter BMD, Intertrochanter BMD, Wards triangle BMD, Total spine BMD, L1 BMD, L2 BMD, L3 BMD, L4 BMD.

Exposure: BMI (kg/m^2^)

Adjust for: Gender, Race, Age, Education level, Ratio of family income to poverty, Smoked at least 100 cigarettes in life, Moderate work activity, Albumin refrigerated serum, Globulin, Glucose refrigerated serum, Cholesterol refrigerated serum, Cholesterol Triglycerides, Standing Height, Arm Circumference, Waist Circumference.

*LRT* test: Log-likelihood ratio test.

**Supplementary Table 4.** Saturation effect analysis of BMI (kg/m^2^) on BMD (g/cm^2^) subgrouped by age

| Outcome | Age(Years) categorical | Model: Saturation effect analysis | | | | *LRT* test |
| --- | --- | --- | --- | --- | --- | --- |
|  |  | BMI Turning point(K), kg/m^2^ | < K, effect 1 | > K , effect 2 | effect 2 - 1 |  |
| Total femur BMD | <=60 | 26.47 | 0.025 (0.023, 0.027) <0.0001 | 0.007 (0.006, 0.007) <0.0001 | -0.018 (-0.021, -0.015)<0.0001 | <0.001 |
|  | >60, <=70 | 28.25 | 0.020 (0.018, 0.022) <0.0001 | 0.005 (0.004, 0.006) <0.0001 | -0.015 (-0.018, -0.013)<0.0001 | <0.001 |
|  | >70 | 26.60 | 0.022 (0.020, 0.025) <0.0001 | 0.006 (0.005, 0.008) <0.0001 | -0.016 (-0.020, -0.013)<0.0001 | <0.001 |
| Femoral neck BMD | <=60 | 26.28 | 0.017 (0.015, 0.019) <0.0001 | 0.007 (0.006, 0.007) <0.0001 | -0.010 (-0.013, -0.008) <0.0001 | <0.001 |
|  | >60, <=70 | 31.76 | 0.013 (0.012, 0.014) <0.0001 | 0.000 (-0.001, 0.002) 0.6889 | -0.012 (-0.015, -0.010) <0.0001 | <0.001 |
|  | >70 | 25.29 | 0.018 (0.015, 0.020) <0.0001 | 0.006 (0.004, 0.007) <0.0001 | -0.012 (-0.015, -0.009) <0.0001 | <0.001 |
| Trochanter BMD | <=60 | 26.55 | 0.019 (0.017, 0.021) <0.0001 | 0.005 (0.004, 0.006) <0.0001 | -0.014 (-0.017, -0.012) <0.0001 | <0.001 |
|  | >60, <=70 | 28.21 | 0.017 (0.015, 0.019) <0.0001 | 0.003 (0.002, 0.004) <0.0001 | -0.014 (-0.016, -0.011) <0.0001 | <0.001 |
|  | >70 | 25.86 | 0.018 (0.016, 0.021) <0.0001 | 0.005 (0.003, 0.006) <0.0001 | -0.014 (-0.017, -0.010) <0.0001 | <0.001 |
| Intertrochanter BMD | <=60 | 26.27 | 0.030 (0.027, 0.033) <0.0001 | 0.007 (0.006, 0.009) <0.0001 | -0.023 (-0.026, -0.019) <0.0001 | <0.001 |
|  | >60, <=70 | 28.21 | 0.024 (0.021, 0.026) <0.0001 | 0.006 (0.004, 0.008) <0.0001 | -0.018 (-0.021, -0.014) <0.0001 | <0.001 |
|  | >70 | 26.60 | 0.028 (0.025, 0.031) <0.0001 | 0.008 (0.006, 0.010) <0.0001 | -0.020 (-0.024, -0.016) <0.0001 | <0.001 |
| Wards triangle BMD | <=60 | 26.13 | 0.013 (0.011, 0.016) <0.0001 | 0.006 (0.005, 0.007) <0.0001 | -0.008 (-0.011, -0.005) <0.0001 | <0.001 |
|  | >60, <=70 | 31.80 | 0.010 (0.009, 0.012) <0.0001 | 0.001 (-0.001, 0.003) 0.1958 | -0.009 (-0.012, -0.006) <0.0001 | <0.001 |
|  | >70 | 29.47 | 0.011 (0.009, 0.012) <0.0001 | 0.002 (-0.000, 0.004) 0.0690 | -0.009 (-0.012, -0.006) <0.0001 | <0.001 |
| Total spine BMD | <=60 | 27.00 | 0.017 (0.014, 0.019) <0.0001 | 0.005 (0.003, 0.006) <0.0001 | -0.012 (-0.016, -0.009) <0.0001 | <0.001 |
|  | >60, <=70 | 31.80 | 0.015 (0.013, 0.017) <0.0001 | 0.002 (-0.001, 0.005) 0.2425 | -0.013 (-0.018, -0.009) <0.0001 | <0.001 |
|  | >70 | 24.81 | 0.025 (0.019, 0.030) <0.0001 | 0.008 (0.005, 0.010) <0.0001 | -0.017 (-0.024, -0.010) <0.0001 | <0.001 |
| L1 BMD | <=60 | 26.28 | 0.019 (0.016, 0.021) <0.0001 | 0.006 (0.005, 0.007) <0.0001 | -0.013 (-0.016, -0.010) <0.0001 | <0.001 |
|  | >60, <=70 | 32.52 | 0.017 (0.015, 0.018) <0.0001 | 0.002 (-0.001, 0.004) 0.2500 | -0.015 (-0.019, -0.011) <0.0001 | <0.001 |
|  | >70 | 25.2 | 0.025 (0.020, 0.029) <0.0001 | 0.009 (0.007, 0.011) <0.0001 | -0.016 (-0.021, -0.010) <0.0001 | <0.001 |
| L2 BMD | <=60 | 29.78 | 0.014 (0.013, 0.016) <0.0001 | 0.002 (0.001, 0.004) 0.0037 | -0.012 (-0.015, -0.009) <0.0001 | <0.001 |
|  | >60, <=70 | 32.14 | 0.015 (0.014, 0.017) <0.0001 | 0.001 (-0.002, 0.004) 0.4534 | -0.014 (-0.018, -0.010) <0.0001 | <0.001 |
|  | >70 | 25.20 | 0.024 (0.019, 0.028) <0.0001 | 0.007 (0.005, 0.009) <0.0001 | -0.016 (-0.022, -0.011) <0.0001 | <0.001 |
| L3 BMD | <=60 | 26.43 | 0.017 (0.014, 0.020) <0.0001 | 0.005 (0.004, 0.006) <0.0001 | -0.012 (-0.016, -0.009) <0.0001 | <0.001 |
|  | >60, <=70 | 32.76 | 0.013 (0.011, 0.015) <0.0001 | 0.000 (-0.003, 0.003) 0.7975 | -0.013 (-0.017, -0.009) <0.0001 | <0.001 |
|  | >70 | 24.74 | 0.022 (0.017, 0.027) <0.0001 | 0.006 (0.004, 0.008) <0.0001 | -0.016 (-0.022, -0.009) <0.0001 | <0.001 |
| L4 BMD | <=60 | 26.25 | 0.017 (0.014, 0.020) <0.0001 | 0.005 (0.004, 0.007) <0.0001 | -0.011 (-0.015, -0.008) <0.0001 | <0.001 |
|  | >60, <=70 | 31.70 | 0.014 (0.012, 0.016) <0.0001 | 0.002 (-0.001, 0.004) 0.2520 | -0.013 (-0.017, -0.009) <0.0001 | <0.001 |
|  | >70 | 24.95 | 0.022 (0.017, 0.027) <0.0001 | 0.005 (0.003, 0.007) <0.0001 | -0.017 (-0.023, -0.010) <0.0001 | <0.001 |

Results in the table: *β* (*95% CI*) *P*-value

Outcome: Total femur BMD, Femoral neck BMD, Femoral neck BMD, Trochanter BMD, Intertrochanter BMD, Wards triangle BMD, Total spine BMD, L1 BMD, L2 BMD, L3 BMD, L4 BMD.

Exposure: BMI (kg/m^2^)

Adjust for: Gender, Race, Age, Education level, Ratio of family income to poverty, Smoked at least 100 cigarettes in life, Moderate work activity, Albumin refrigerated serum, Globulin, Glucose refrigerated serum, Cholesterol refrigerated serum, Cholesterol Triglycerides, Standing Height, Arm Circumference, Waist Circumference.

*LRT* test: Log-likelihood ratio test.

**Supplementary Table 5.** Saturation effect analysis of BMI (kg/m^2^) on BMD (g/cm^2^) subgrouped by race

| Outcome | Race | Model: Saturation effect analysis | | | | *LRT* test |
| --- | --- | --- | --- | --- | --- | --- |
|  |  | BMI Turning point(K), kg/m^2^ | < K, effect 1 | > K , effect 2 | effect 2 - 1 |  |
| Total femur BMD | Mexican American | 27.29 | 0.026 (0.021, 0.030) <0.0001 | 0.005 (0.003, 0.006) <0.0001 | -0.021 (-0.027, -0.016) <0.0001 | <0.001 |
|  | Other Hispanic | 26.80 | 0.022 (0.016, 0.027) <0.0001 | 0.009 (0.006, 0.011) <0.0001 | -0.013 (-0.020, -0.007) 0.0001 | <0.001 |
|  | Non-Hispanic White | 26.60 | 0.024 (0.022, 0.026) <0.0001 | 0.007 (0.007, 0.008) <0.0001 | -0.016 (-0.019, -0.014) <0.0001 | <0.001 |
|  | Non-Hispanic Black | 26.80 | 0.018 (0.015, 0.022) <0.0001 | 0.005 (0.004, 0.006) <0.0001 | -0.013 (-0.018, -0.009) <0.0001 | <0.001 |
|  | Other Race | 25.20 | 0.029 (0.024, 0.034) <0.0001 | 0.006 (0.004, 0.009) <0.0001 | -0.022 (-0.029, -0.016) <0.0001 | <0.001 |
| Femoral neck BMD | Mexican American | 35.48 | 0.018 (0.014, 0.021) <0.0001 | 0.007 (0.003, 0.011) 0.0006 | -0.010 (-0.015, -0.006) <0.0001 | <0.001 |
|  | Other Hispanic | 29.49 | 0.016 (0.011, 0.021) <0.0001 | 0.013 (0.009, 0.017) <0.0001 | -0.003 (-0.008, 0.002) 0.2087 | 0.204 |
|  | Non-Hispanic White | 29.60 | 0.019 (0.017, 0.021) <0.0001 | 0.012 (0.011, 0.014) <0.0001 | -0.006 (-0.008, -0.004) <0.0001 | <0.001 |
|  | Non-Hispanic Black | 29.01 | 0.020 (0.017, 0.024) <0.0001 | 0.011 (0.008, 0.014) <0.0001 | -0.009 (-0.013, -0.006) <0.0001 | <0.001 |
|  | Other Race | 24.92 | 0.028 (0.021, 0.035) <0.0001 | 0.011 (0.007, 0.015) <0.0001 | -0.017 (-0.023, -0.011) <0.0001 | <0.001 |
| Trochanter BMD | Mexican American | 35.10 | 0.017 (0.014, 0.021) <0.0001 | 0.005 (0.001, 0.009) 0.0123 | -0.012 (-0.017, -0.008) <0.0001 | <0.001 |
|  | Other Hispanic | 36.32 | 0.013 (0.009, 0.017) <0.0001 | 0.006 (-0.000, 0.012) 0.0639 | -0.007 (-0.014, -0.001) 0.0323 | 0.031 |
|  | Non-Hispanic White | 26.38 | 0.021 (0.018, 0.023) <0.0001 | 0.012 (0.011, 0.014) <0.0001 | -0.008 (-0.011, -0.006) <0.0001 | <0.001 |
|  | Non-Hispanic Black | 27.31 | 0.018 (0.014, 0.022) <0.0001 | 0.008 (0.006, 0.011) <0.0001 | -0.010 (-0.013, -0.006) <0.0001 | <0.001 |
|  | Other Race | 27.14 | 0.022 (0.018, 0.027) <0.0001 | 0.009 (0.005, 0.013) <0.0001 | -0.013 (-0.018, -0.008) <0.0001 | <0.001 |
| Intertrochanter BMD | Mexican American | 35.90 | 0.023 (0.019, 0.028) <0.0001 | 0.006 (-0.000, 0.011) 0.0564 | -0.018 (-0.024, -0.012) <0.0001 | <0.001 |
|  | Other Hispanic | 33.40 | 0.018 (0.013, 0.024) <0.0001 | 0.008 (0.001, 0.015) 0.0192 | -0.011 (-0.018, -0.004) 0.0033 | 0.003 |
|  | Non-Hispanic White | 26.20 | 0.029 (0.026, 0.032) <0.0001 | 0.016 (0.013, 0.018) <0.0001 | -0.013 (-0.016, -0.010) <0.0001 | <0.001 |
|  | Non-Hispanic Black | 28.94 | 0.025 (0.020, 0.029) <0.0001 | 0.011 (0.007, 0.014) <0.0001 | -0.014 (-0.018, -0.010) <0.0001 | <0.001 |
|  | Other Race | 24.92 | 0.041 (0.033, 0.050) <0.0001 | 0.013 (0.007, 0.018) <0.0001 | -0.029 (-0.037, -0.021) <0.0001 | <0.001 |
| Wards triangle BMD | Mexican American | 27.15 | 0.022 (0.016, 0.029) <0.0001 | 0.013 (0.009, 0.017) <0.0001 | -0.009 (-0.015, -0.004) 0.0016 | 0.001 |
|  | Other Hispanic | 37.90 | 0.014 (0.009, 0.019) <0.0001 | 0.004 (-0.006, 0.014) 0.4201 | -0.010 (-0.020, -0.000) 0.0476 | 0.045 |
|  | Non-Hispanic White | 29.90 | 0.018 (0.015, 0.020) <0.0001 | 0.011 (0.009, 0.013) <0.0001 | -0.007 (-0.009, -0.005) <0.0001 | <0.001 |
|  | Non-Hispanic Black | 29.12 | 0.020 (0.015, 0.024) <0.0001 | 0.010 (0.006, 0.013) <0.0001 | -0.010 (-0.014, -0.006) <0.0001 | <0.001 |
|  | Other Race | 22.36 | 0.041 (0.029, 0.054) <0.0001 | 0.009 (0.003, 0.014) 0.0012 | -0.033 (-0.045, -0.020) <0.0001 | <0.001 |
| Total spine BMD | Mexican American | 22.44 | 0.029 (0.013, 0.046) 0.0005 | 0.008 (0.006, 0.010) <0.0001 | -0.021 (-0.038, -0.004) 0.0138 | 0.014 |
|  | Other Hispanic | 22.79 | 0.031 (0.012, 0.050) 0.0019 | 0.006 (0.004, 0.009) <0.0001 | -0.025 (-0.045, -0.004) 0.0181 | 0.018 |
|  | Non-Hispanic White | 27.10 | 0.018 (0.016, 0.021) <0.0001 | 0.005 (0.004, 0.007) <0.0001 | -0.013 (-0.017, -0.010) <0.0001 | <0.001 |
|  | Non-Hispanic Black | 37.60 | 0.008 (0.006, 0.010) <0.0001 | -0.005 (-0.011, 0.000) 0.0683 | -0.013 (-0.020, -0.007) <0.0001 | <0.001 |
|  | Other Race | 25.14 | 0.028 (0.022, 0.034) <0.0001 | 0.002 (-0.002, 0.005) 0.3946 | -0.027 (-0.035, -0.018) <0.0001 | <0.001 |
| L1 BMD | Mexican American | 24.80 | 0.025 (0.016, 0.035) <0.0001 | 0.014 (0.010, 0.018) <0.0001 | -0.011 (-0.020, -0.002) 0.0167 | 0.016 |
|  | Other Hispanic | 22.79 | 0.027 (0.006, 0.047) 0.0109 | 0.010 (0.004, 0.015) 0.0007 | -0.017 (-0.037, 0.004) 0.1074 | 0.103 |
|  | Non-Hispanic White | 31.86 | 0.016 (0.013, 0.019) <0.0001 | 0.007 (0.005, 0.010) <0.0001 | -0.009 (-0.011, -0.006) <0.0001 | <0.001 |
|  | Non-Hispanic Black | 27.30 | 0.022 (0.017, 0.028) <0.0001 | 0.010 (0.007, 0.013) <0.0001 | -0.012 (-0.017, -0.007) <0.0001 | <0.001 |
|  | Other Race | 22.36 | 0.042 (0.029, 0.054) <0.0001 | 0.010 (0.005, 0.016) <0.0001 | -0.031 (-0.043, -0.019) <0.0001 | <0.001 |
| L2 BMD | Mexican American | 24.30 | 0.030 (0.019, 0.041) <0.0001 | 0.015 (0.010, 0.019) <0.0001 | -0.015 (-0.026, -0.005) 0.0052 | 0.005 |
|  | Other Hispanic | 22.79 | 0.043 (0.021, 0.066) 0.0001 | 0.012 (0.006, 0.018) <0.0001 | -0.031 (-0.053, -0.009) 0.0061 | 0.006 |
|  | Non-Hispanic White | 30.94 | 0.019 (0.016, 0.022) <0.0001 | 0.009 (0.006, 0.012) <0.0001 | -0.010 (-0.013, -0.007) <0.0001 | <0.001 |
|  | Non-Hispanic Black | 29.72 | 0.019 (0.014, 0.024) <0.0001 | 0.008 (0.004, 0.011) <0.0001 | -0.011 (-0.016, -0.007) <0.0001 | <0.001 |
|  | Other Race | 22.35 | 0.045 (0.031, 0.058) <0.0001 | 0.011 (0.005, 0.016) <0.0001 | -0.034 (-0.047, -0.021) <0.0001 | <0.001 |
| L3 BMD | Mexican American | 23.87 | 0.026 (0.013, 0.040) <0.0001 | 0.016 (0.012, 0.021) <0.0001 | -0.010 (-0.023, 0.002) 0.1146 | 0.112 |
|  | Other Hispanic | 22.79 | 0.045 (0.024, 0.067) <0.0001 | 0.011 (0.005, 0.017) 0.0004 | -0.034 (-0.056, -0.012) 0.0022 | 0.002 |
|  | Non-Hispanic White | 32.90 | 0.018 (0.015, 0.021) <0.0001 | 0.008 (0.005, 0.011) <0.0001 | -0.010 (-0.013, -0.006) <0.0001 | <0.001 |
|  | Non-Hispanic Black | 32.27 | 0.017 (0.012, 0.022) <0.0001 | 0.007 (0.003, 0.011) 0.0014 | -0.010 (-0.015, -0.006) <0.0001 | <0.001 |
|  | Other Race | 24.90 | 0.034 (0.024, 0.044) <0.0001 | 0.008 (0.002, 0.014) 0.0117 | -0.026 (-0.035, -0.017) <0.0001 | <0.001 |
| L4 BMD | Mexican American | 23.33 | 0.032 (0.015, 0.049) 0.0003 | 0.022 (0.017, 0.027) <0.0001 | -0.010 (-0.027, 0.007) 0.2448 | 0.241 |
|  | Other Hispanic | 22.79 | 0.062 (0.040, 0.085) <0.0001 | 0.013 (0.007, 0.020) <0.0001 | -0.049 (-0.071, -0.027) <0.0001 | <0.001 |
|  | Non-Hispanic White | 30.78 | 0.020 (0.017, 0.023) <0.0001 | 0.012 (0.009, 0.015) <0.0001 | -0.008 (-0.011, -0.005) <0.0001 | <0.001 |
|  | Non-Hispanic Black | 32.34 | 0.017 (0.012, 0.022) <0.0001 | 0.005 (0.001, 0.010) 0.0188 | -0.012 (-0.016, -0.007) <0.0001 | <0.001 |
|  | Other Race | 24.27 | 0.041 (0.030, 0.052) <0.0001 | 0.006 (-0.000, 0.012) 0.0693 | -0.035 (-0.045, -0.025) <0.0001 | <0.001 |

Results in the table: *β* (*95% CI*) *P*-value

Outcome: Total femur BMD, Femoral neck BMD, Femoral neck BMD, Trochanter BMD, Intertrochanter BMD, Wards triangle BMD, Total spine BMD, L1 BMD, L2 BMD, L3 BMD, L4 BMD.

Exposure: BMI (kg/m^2^)

Adjust for: Gender, Race, Age, Education level, Ratio of family income to poverty, Smoked at least 100 cigarettes in life, Moderate work activity, Albumin refrigerated serum, Globulin, Glucose refrigerated serum, Cholesterol refrigerated serum, Cholesterol Triglycerides, Standing Height, Arm Circumference, Waist Circumference.

*LRT* test: Log-likelihood ratio test.
